# Astragalus polyphenols attenuates doxorubicin-induced cardiotoxicity by activating the PI3K/AKT/NRF2 pathway

**DOI:** 10.1371/journal.pone.0319067

**Published:** 2025-02-25

**Authors:** Xueyang Bai, Hua Wei, Gangqiong Liu, Ling Li

**Affiliations:** 1 Department of Cardiology, The First Affiliated Hospital of Zhengzhou University, Zhengzhou, Henan, China; 2 Department of Cardiology, Hami Central Hospital, Hami, Xinjiang, China; University of Pennsylvania, UNITED STATES OF AMERICA

## Abstract

**Background:**

Doxorubicin (DOX) is a powerful chemotherapeutic agent commonly employed in cancer treatment. However, its clinical utility is constrained by dose-dependent cardiotoxicity, which can result in heart failure and sudden cardiac death. The molecular mechanisms of DOX-induced cardiotoxicity (DIC) include oxidative stress, mitochondrial dysfunction, and the activation of cell death pathways, including ferroptosis. There is an urgent need for effective therapeutic strategies to mitigate DIC.

**Methods:**

This study investigates the cardioprotective effects of Astragalus Polyphenols (ASP), a bioactive compound extracted from Astragalus membranaceus. In the context of DIC, we utilized AC16 and H9C2 cardiomyocytes to establish a DIC model and assessed the effects of ASP on cell viability, oxidative stress, mitochondrial function, and the PI3K/AKT/NRF2 signaling pathway. The expression of atrial natriuretic peptide (ANP) and brain natriuretic peptide (BNP), markers of cardiac injury, was also evaluated.

**Results:**

ASP treatment significantly reversed DOX-induced reductions in cell viability and mitochondrial membrane potential (MMP) while also decreasing the levels of reactive oxygen species (ROS). Additionally, ASP also downregulated the expression of ANP and BNP, indicating a protective effect on cardiomyocytes. Furthermore, ASP activated the PI3K/AKT/NRF2 pathway, which was suppressed by DOX. Inhibition of this pathway using LY294002 and ML385 abolishes the protective effects of ASP, suggesting that ASP mediates its effects through the PI3K/AKT/NRF2 signaling axis.

**Conclusion:**

ASP exhibits a protective effect against DOX-induced cardiotoxicity by regulating the PI3K/AKT/NRF2 pathway to reduce oxidative stress and preserve mitochondrial function. These findings suggest that ASP may serve as a potential therapeutic agent to alleviate DIC. Our results provide a novel strategy to protect the heart in patients undergoing DOX chemotherapy.

## Introduction

Heart failure disease is caused by different reasons that impair the heart’s ability to pump blood for the whole body effectively, which is one of the major global health problems. One of the main causes is DIC, a dark effect of DOX, which is widely used in cancer treatment as a chemotherapeutic agent [1,2]. While DOX is highly effective against various tumors, its use is limited by the risk of cardiotoxicity, which can lead to heart failure and sudden cardiac death [1,3].

DIC has three main characteristics, including severe oxidative stress, mitochondrial dysfunction, and cell death pathway activation, such as ferroptosis, autophagy, and apoptosis [4–6]. When cell death pathways are activated, it can lead to irreversible cardiac damage and heart failure if not treated in time [7]. Ferroptosis is a major reason for DOX-induced cell death. Nowadays, in clinical therapeutic reagants for alleviative DIC, only dexzorexen has been approved for the treatment. However, because it provides limited protection and may come with serious myelosuppressive side effects, it was urgent to find novel therapeutic approaches [8].

Astragalus membranaceus, which is one of the traditional Chinese medicinal herbs, has been used to treat cardiovascular diseases for a long time [9,10]. Its compounds, such as Astragaloside IV, and Astragalus polysaccharide, have shown the potential ability to counteract oxidative stress and maintain cardiovascular homeostasis by regular cellular pathways [9]. In previous studies, Astragaloside IV could attenuate DIC by modulating SIRT1 [11]. In another study, APS could protect the myocardium by restoring autophagy flow [12]. As one of the most important extracts of Astragalus membranaceus, ASP also has an antioxidant effect, and in the study of Qingfeng Yan et al., ASP has the effect of protecting mitochondria and can significantly improve the mitochondrial membrane potential (MMP) [13]. These studies revealed that ASP might be promising in rescuing cardiotoxicity caused by DOX.

PI3K/AKT pathway is indispensable for cell survival and is involved in cellular energy metabolism and cellular defense against oxidative stress [14]. Similarly, PI3K is also important in regulating the ferroptosis-related pathway. Nuclear factor erythroid 2-related factor 2 (NRF2) is one of the most important downstream targets of PI3K/AKT [15], which is strongly correlated with ferroptosis [4,16]. Whether ASP could recover mitochondrial damage and alleviate oxidative stress in the DIC model is still to be fully understood. We hypothesize that ASP may reduce oxidative stress, preserve mitochondrial function, and regulate cardiac injury. Additionally, we aimed to explore the mechanisms by which ASP may protect cardiomyocytes from DOX-induced damage. This may provide a possible new treatment option for patients undergoing chemotherapy to alleviate anthracycline cardiotoxicity.

## Methods

### Cell line and culture

In this experiment, we used the AC16 and H9C2 cell lines to construct an in vitro DIC model. The AC16 and H9C2 cells were purchased from Wuhan Pricella Biotechnology (CL-0790, CL-0089, Wuhan, China). Doxorubicin, Astragalus polyphenols, LY294002 (PI3K inhibitor), and ML385 (Nrf2 inhibitor) were obtained from Selleck Chemicals(Houston, TX). AC16 and H9C2 cells were maintained in DMEM (Gibco, NY, USA) with 10% fetal bovine serum (FBS, Vazyme, Nanjing, China), 100 units/ml penicillin, and 100 μg/ml streptomycin at 37˚C with 5% CO2. In this experiment, 0.5 μM DOX was used to treat AC16 and H9C2 cells to establish the DIC model when AC16 and H9C2 cells were at 70% confluence for 24h. For the DOX+ASP group, different doses of ASP (1,10,20,30, and 40 μM) were used to treat AC16 and H9C2 cells for 24h with DOX. To inhibit the PI3K/AKT pathway, the DOX+ASP+LY294002 group was pre-treated with 20μM LY294002 [17] and then incubated with DOX and ASP for 24h. For inhibition of the Nrf2 pathway, 5μM ML385 was co-treated with DOX and ASP in the DOX+ASP+ML385 group for 24h.

### Cell counting kit-8 assay

Cell Counting Kit-8 (CCK-8) assay was purchased from Beyotime Biotechnology (C0038, Shanghai, China). CCK-8 assay was used to assess cell proliferation. We seeded AC16 and H9C2 cells into a 96-well plate at 3 × 10^3^ cell/well density at 37° C with 5% CO2 for 24h. After the cells had undergone the abovementioned treatments, we added 10 µ L of CCK-8 reagent to each well for 2h of incubation. Then, absorbance at 450 nm was measured with a microplate reader (1410101, Thermo Fisher Scientific, USA).

### Measurement of mitochondrial membrane potential (ψ): JC-1 staining

JC-1 staining kit (C2003S, Beyotime Biotechnology, Shanghai, China) was used to measure the changes in mitochondrial membrane potential (MMP). Different groups of cells were treated with JC-1 (10 μg/mL) at 37° C with 5% CO2 for 25 minutes. Then, the medium was removed, and the cells were washed three times using PBS. JC-1 fluorescence was determined by fluorescence microscopy.

### Measurement of ROS

The levels of total ROS in AC16 cells were evaluated using a dichloro-dihydro-fluorescein diacetate (DCFH-DA) staining kit (S0033S, Beyotime Biotechnology, Shanghai, China). After being treated with DCFH-DA for 30 minutes, the cells were collected for flow cytometry. The data were analyzed using the FlowJo software(FlowJo V10.8.1). For the analysis, three samples from each experimental group were selected, and the mean fluorescence intensity (FITC value) of the control group was calculated. Subsequently, the data from each sample were normalized by dividing their values by the mean fluorescence intensity of the control group. The normalized data were then subjected to statistical analysis using GraphPad Prism 10.0.

### Western blot

The cellular total protein was extracted using RIPA lysis buffer with protease and phosphatase inhibitors. After centrifugation, the total protein was mixed with 5X loading buffer and heated to 95°C for 10 minutes. Equal proteins were separated using electrophoresis and then transferred onto the PVDF membrane. After being transferred, the PVDF membranes were blocked with 5% skimmed milk dissolved in TBST for 2h at 25°C temperature, followed by the membranes incubated with specific antibodies for 15h at 4°C. After this, the membranes were washed 3 times with TBST and then incubated with the secondary antibody for 2h at room temperature. Finally, the membranes reacted with enhanced chemiluminescence (ECL), and the grey value was analyzed using ImageJ software.

### Quantitative real-time PCR (qRT-PCR)

Total RNA was collected from cells using the RNeasy fast extract Kit (AC0202, Sparkjade, Shandong, China). First-strand complementary DNA (cDNA) was synthesized from 0.5 μg of total RNA using the High-Capacity cDNA Reverse Transcription Kit (Vazyme, Nanjing, China) in a 20 μL reaction volume, following the manufacturer’s instructions. qPCR was performed using the PowerUp SYBR Green Master Mix (Vazyme, Nanjing, China) on a QuantStudio 7 Flex Real-Time PCR system. Primers for ANP, BNP, and the housekeeping gene GAPDH were designed using Primer-BLAST (NCBI) and are listed in supplementary S1 Table. Data are presented as the mean ±  SEM from three independent experiments performed in triplicate. Statistical analysis was performed using a one-way ANOVA followed by Tukey’s post-hoc test, with p <  0.05 considered statistically significant.

### Statistics

All the results were analyzed by using GraphPad Prism 10. At least three biological replicates for each experiment are presented as mean±SD. Comparisons between the two groups were using the student’s t-test, among multiple groups were compared with one-way ANOVA. Sample sizes were presented in every figure legend. P < 0.05 was considered statistically significant.

### Ethical statement

The AC16 and H9C2 cell lines used in this study were obtained from commercial sources (CL-0790, CL-0089, Wuhan Pricella Biotechnology, Wuhan, China) and were found to be free of mycoplasma contamination.

## Results

### ASP alleviates DOX-induced cardiotoxicity and oxidative stress in AC16 and H9C2 cells

**Fig 1:**
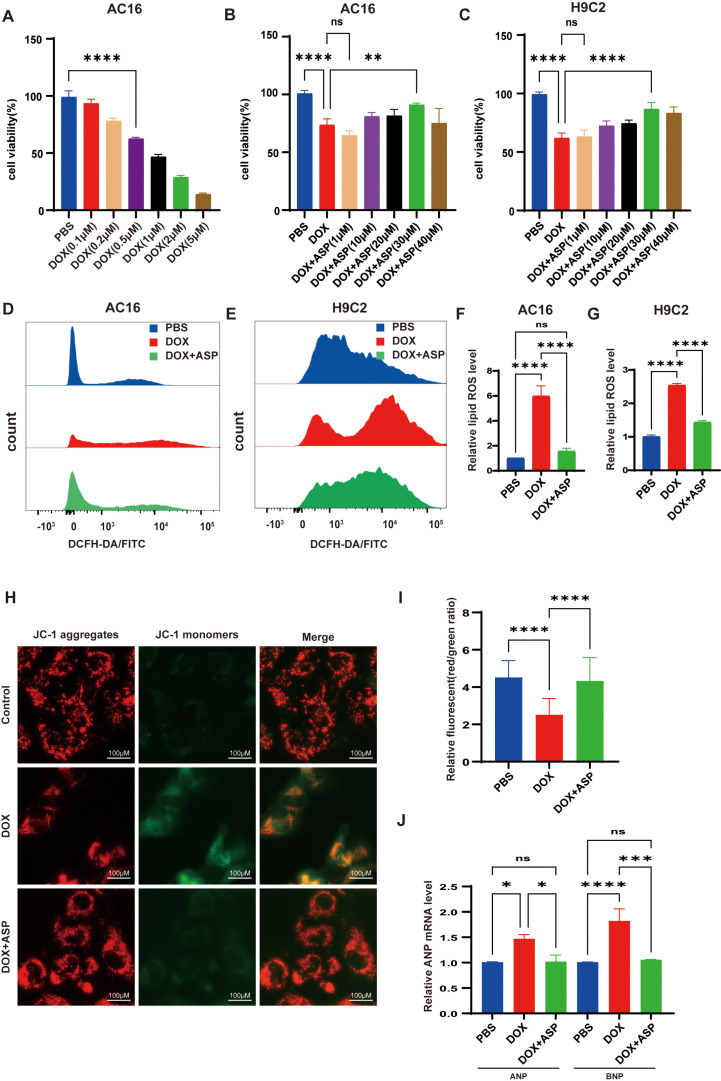
ASP alleviates DOX-induced cardiotoxicity and oxidative stress in AC16 and H9C2 cells. (A) The CCK-8 assay was utilized to assess cell viability in DOX-treated AC16 cells (n = 4); CCK-8 assay for detecting the protective effect of ASP on DOX-injured AC16 (B) and H9C2 (C) cells (n = 4); Flow cytometry was used to detected DCFH-DA/FITC along with statistical analysis in AC16 (D&F) and H9C2 (E&G) cells (n = 3); (H&I) The effects of ASP on the reduction of MMP potential induced by DOX in AC16 cells were measured (red/green ratio) (Scale bar =  100 μm, n = 150); (J) Quantitative q-PCR analysis of ANP and BNP mRNA expression in AC16 and H9C2 cells was conducted (n = 3); For the data presented above, one-way ANOVA (Tukey post-test), means ±  SD. P >  0.05 indicates nonsignificant (ns), *  P <  0.05, ** P <  0.01, *** P <  0.001, **** P <  0.0001.

In the present study, we conducted an initial evaluation of the effects of various concentrations of doxorubicin (DOX) (0.1, 0.2, 0.5, 1, 2, and 5 μM) on the viability of AC16 cells utilizing the CCK-8 assay kit. The results indicated that after 24-hour exposure to DOX, the cell viability in the 0.5 μM treatment group was approximately 62.0% relative to the control group (Fig 1A). Consequently, we selected 0.5 μM DOX for subsequent in vitro modeling of doxorubicin-induced cardiotoxicity (DIC). It was observed that this concentration caused significant damage to both AC16 and H9C2 cells, as shown in S1 Fig. This concentration has also been employed in previous studies [18]. To investigate the protective effects of ASP against DOX-induced cellular damage, we treated AC16 and H9C2 cells with varying concentrations of ASP (1, 10, 20, 30, and 40 μM) in conjunction with 0.5 μM DOX for 24 hours. Then, the CCK8 assay was subsequently utilized to evaluate the efficacy of ASP in DOX-induced cardiotoxicity. As illustrated in Fig 1B and C, ASP significantly counteracted the reduction in cell viability induced by DOX at a concentration of 30 μM, which we therefore selected as the therapeutic concentration in subsequent experiments. Previous studies have demonstrated that DOX induces the development of oxidative stress in cardiomyocytes and causes mitochondrial damage [2]. To quantify myocardial lipid ROS levels and MMP, we employed DCFA and JC-1 staining techniques to assess oxidative stress and mitochondrial impairment. Our findings demonstrated that DOX significantly increased ROS levels (Fig 1D–G) and decreased MMP (Fig 1H and I); however, the administration of ASP treatment notably mitigated this damage. In addition, ASP significantly reduced DOX-induced elevations in markers of myocardial injury, including atrial natriuretic peptide (ANP) and brain natriuretic peptide (BNP) (Fig 1J). The above results showed that ASP could reverse the myocardial and mitochondrial damage induced by DOX.

### Effects of ASP on PI3K/AKT/Nrf2 pathway in AC16 cells

**Fig 2:**
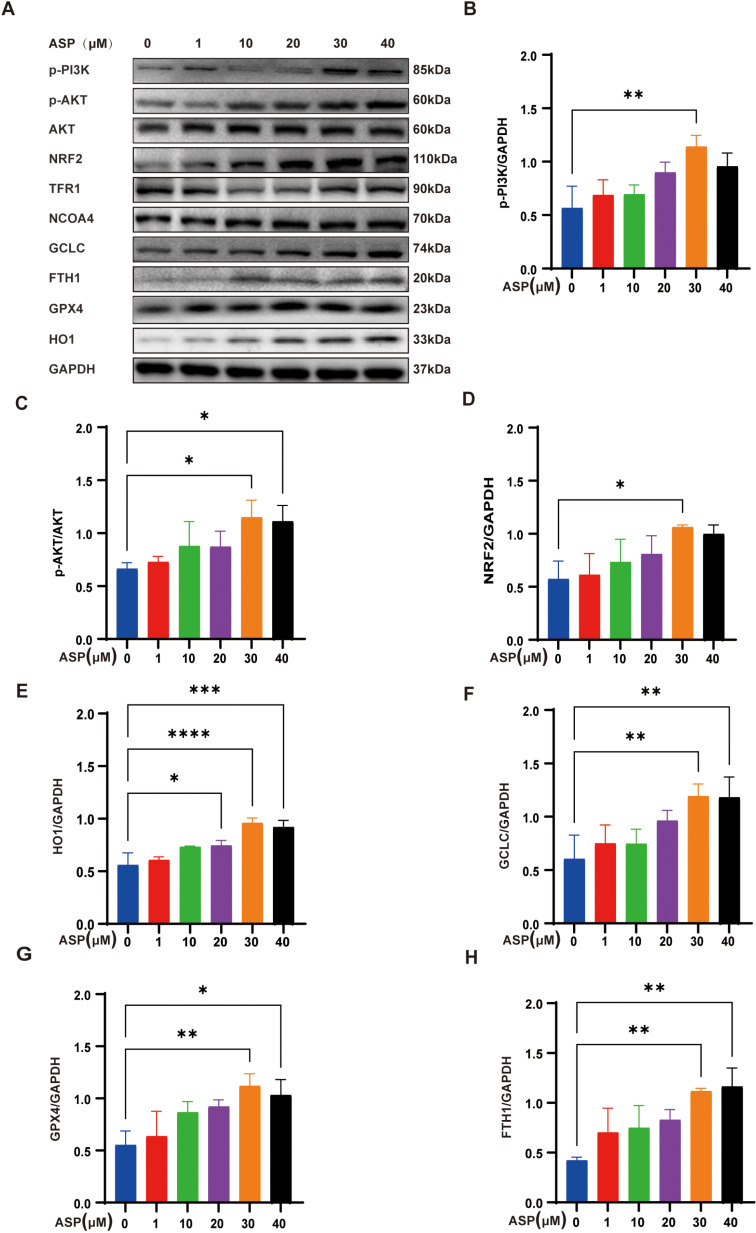
Effects of ASP on PI3K/AKT/Nrf2 pathway in AC16 cells. (A) Representative protein levels of p-PI3K, p-AKT, AKT, Nrf2, and ferroptosis-related signaling pathways in different concentrations of ASP-treated AC16 cells; (B-H) Statistical analysis of relative protein expression shown in Fig 2A (n = 3); One-way ANOVA (Tukey post-test), means ±  SD. P >  0.05, nonsignificant (ns), *  P <  0.05, ** P <  0.01, *** P <  0.001, **** P <  0.0001.

In previous studies, the PI3K/AKT signaling pathway has been identified as crucial for myocardial defense against oxidative stress and DOX-induced ferropotosis [19]. Astragalus membranaceus has been shown to activate both PI3K and Nrf2, with ASP, a key component, potentially providing cardioprotective effects through this signaling pathway [4,19,20]. The findings indicate that the expression levels of p-PI3K^85^, AKT, and p-AKT^473^ are increased in a dose-dependent manner in response to ASP treatment (Fig 2A–C). As depicted in Fig 1D and G, ASP has demonstrated a significant reduction in intracellular ROS. Importantly, NRF2 is recognized as a key gene in the defense against cellular oxidative stress, playing a crucial role in lowering ROS levels [21]. Additionally, it is well-documented that heightened AKT activity contributes to the stabilization of NRF2, thereby reducing its degradation [22,23]. Leveraging these insights, we advanced our investigation to explore NRF2 and its downstream targets. The results indicated that Nrf2 and its downstream genes, such as HO1, GCLC, and GPX4, which play a crucial role in regulating glutathione metabolism and antioxidant responses during ferroptosis, were activated with increasing doses of ASP (Fig 2D–G). Interestingly, the expression of FTH1 also increased in a dose-dependent manner with the increase of ASP concentration, while the expression of NCOA4 did not change significantly. This suggests that the increase in FTH1 may be attributed to the regulation of Nrf2 activation (Fig 2AandH). In addition, there was no significant difference in the expression of the ferroptosis-related iron metabolism gene TFRC1, suggesting that ASP may play an antioxidant effect by regulating Nrf2 and its downstream genes to protect the myocardium from DOX damage(Fig 2A). In summary, ASP can significantly activate the PI3K/AKT/Nrf2 signaling pathway at a concentration of 30 μM.

### Combined treatment of ASP rescues DOX-induced suppression of the PI3K/AKT/Nrf2 pathway in AC16 cells

**Fig 3:**
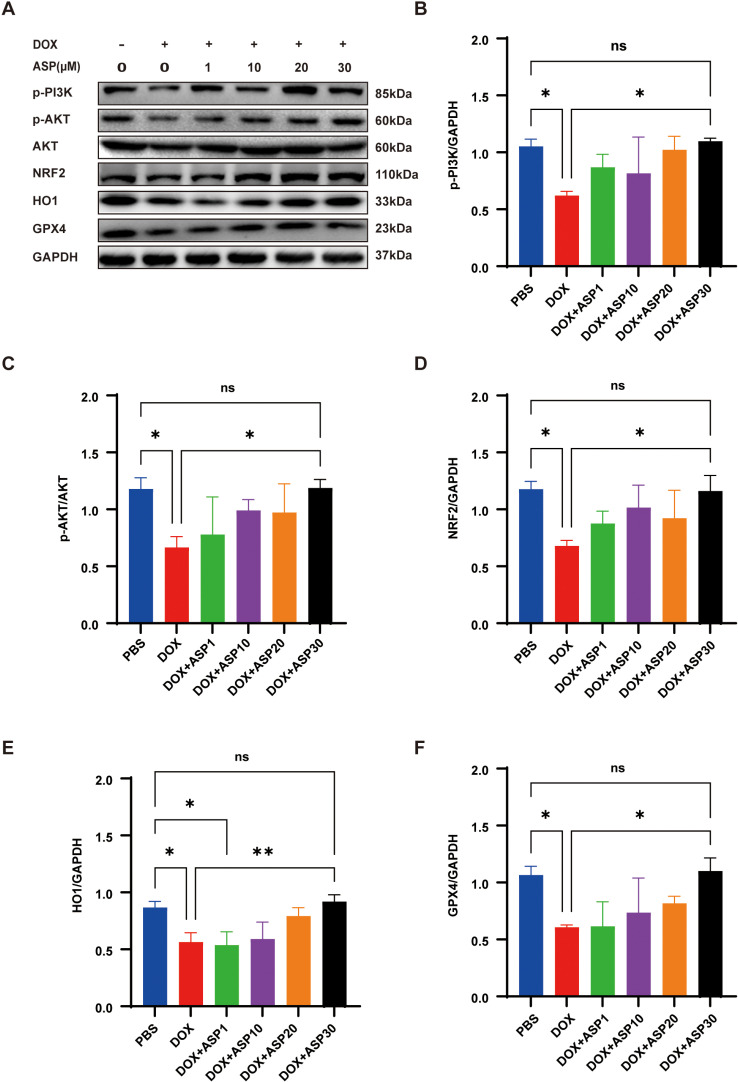
Combined treatment of ASP rescues DOX-induced suppression of the PI3K/AKT/Nrf2 pathway in AC16 cells. (A) The protein levels of p-PI3K, p-AKT, AKT, Nrf2, and the ferroptosis-related signaling pathway in AC16 cells; (B-F) WB analysis demonstrates that ASP restores the expression of p-PI3K, p-AKT, AKT, Nrf2 and the downstream pathway in a dose-dependent manner in DOX-treated cells (n = 3); One-way ANOVA (Tukey post-test), means ±  SD. P >  0.05, nonsignificant (ns), *  P <  0.05, ** P <  0.01, *** P <  0.001, **** P <  0.0001.

Previous studies have demonstrated that DOX can significantly inhibit the PI3K/AKT/Nrf2 pathway [24,25], a finding that has also been confirmed in our experiments. The results of WB analysis indicated that the expression levels of p-PI3K^85^, p-Akt^473^/AKT, Nrf2, and its downstream genes, such as GPX4 and HO1, were significantly reduced following DOX treatment (Fig 3A–F). Although we have shown that ASP can significantly activate the PI3K signaling pathway in the aforementioned study, it remains unclear whether ASP can reverse the depression observed in the DIC model. Therefore, we utilized different concentrations of ASP to intervene in the DIC model. As the concentration of ASP increased, the protein expression levels of p-PI3K^85^, AKT, and p-Akt^473^ recovered from the inhibition caused by DOX (Fig 3BandC). Additionally, the expressions of Nrf2, HO1, and GPX4 were elevated, likely due to the activation of the PI3K/AKT pathway (Fig. 3D-F).

### PI3K inhibition abolishes the protective effect of ASP against DOX-induced cardiac injury and mitochondrial damage

**Fig 4:**
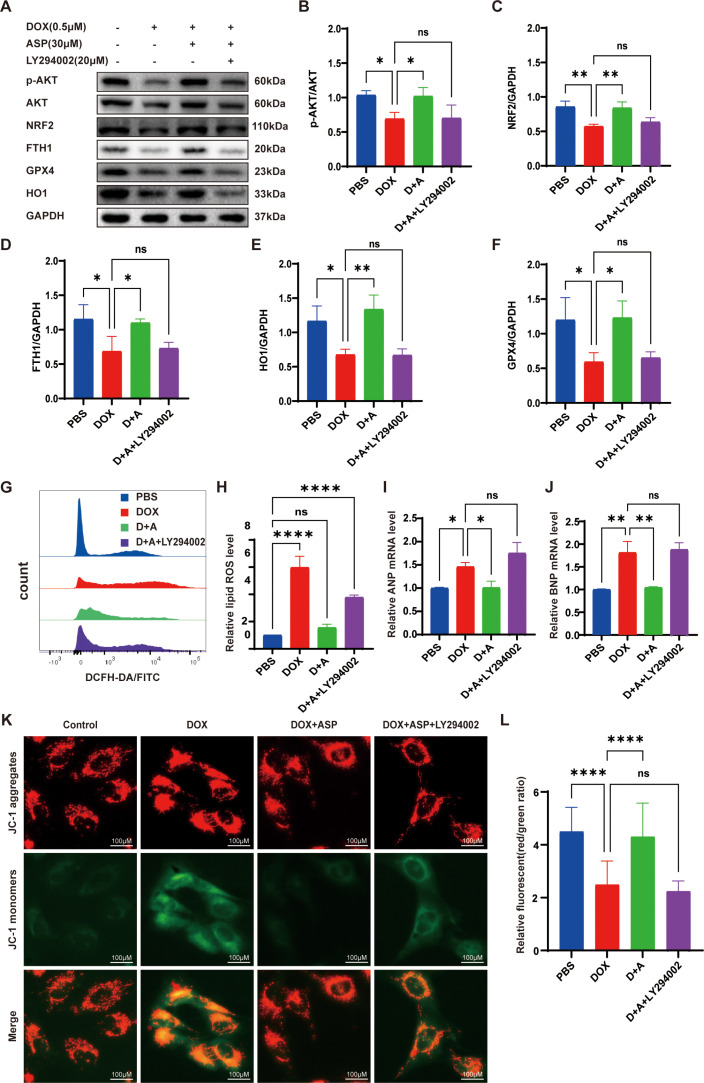
PI3K inhibition abolishes the protective effect of ASP against DOX-induced cardiac injury and mitochondrial damage. (A) Western blots of p-AKT, AKT, Nrf2, and downstream signaling pathways in AC16 cells; (B-F) The statistical results of relative protein expression (n = 3); (G, H) Lipid ROS tested by flow cytometry using DCFH-DA staining (n = 3); (I, J) Quantitative q-PCR analysis of cardiac injury markers, specifically ANP and BNP mRNA expression (n = 3); (K, L) Representative images of JC-1 aggregates (red), JC-1 monomers (green) and merged images of both (merge) (Scale bar =  100 μm), andstatistical analysis of red/green ratio (n = 150); One-way ANOVA (Tukey post-test), means ±  SD. P >  0.05, nonsignificant (ns), *  P <  0.05, ** P <  0.01, *** P <  0.001, **** P <  0.0001.

To further confirm whether ASP functions through the activation of the PI3K pathway, we utilized LY294002 to inhibit the PI3K pathway. LY294002 is a broad-spectrum PI3K inhibitor that significantly reduces the expression of p-AKT^473^. WB analysis indicates that compared to the ASP treatment group, the expression of p-AKT^473^ decreased significantly (Fig 4A and B). Concurrently, the recovery effects of Nrf2, GPX4, HO1, and FTH1 also disappeared (Fig 4C–F). These results demonstrate that increased expression of PI3K/AKT activates Nrf2. When the PI3K pathway was suppressed, as illustrated in Fig 4G and H, LY294002 markedly diminished the antioxidant capacity of ASP, presenting increased lipid ROS levels in AC16 cells. Additionally, LY294002 significantly inhibited the protection effect of ASP on cardiac and mitochondrial injury (Fig. 4I-L)

### ASP protects the AC16 cells from DOX-induced cardiotoxicity via Nrf2 activation

**Fig 5:**
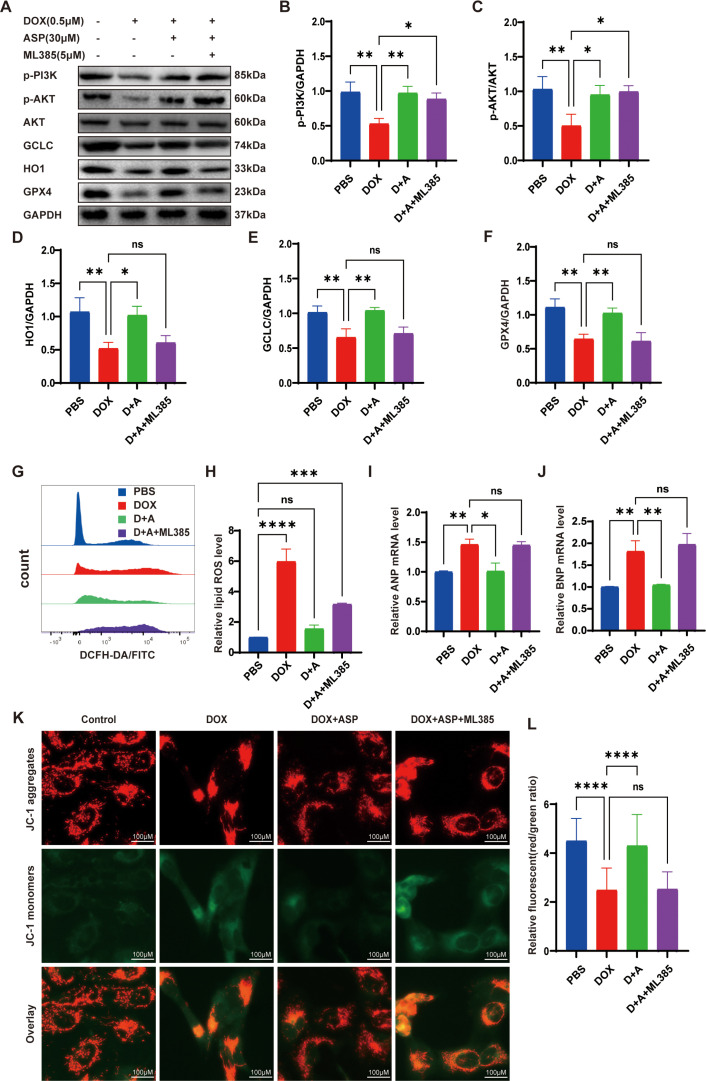
ASP protects the AC16 cells from DOX-induced cardiotoxicity via Nrf2 activation. (A) Representative images of protein expression detected by Western blot of p-PI3K,p-AKT, AKT, and the Nrf2 downstream signaling pathways in AC16 cells; (B, C) ML385 does not affect PI3K/AKT (n = 3); (D-F) The statistical results show that ML385 significantly represses NRF2 activation, leading to a decrease in its downstream gene expression (n = 3); (G, H) Lipid ROS levels (n = 3); (I, J) Quantitative q-PCR analysis of relative ANP and BNP mRNA expression (n = 3); (K, L) Representative images (Scale bar =  100 μm) and statistical analysis of JC-1 (n = 150); One-way ANOVA (Tukey post-test), means ±  SD. P >  0.05, nonsignificant (ns), *  P <  0.05, ** P <  0.01, *** P <  0.001, **** P <  0.0001.

The Nrf2 pathway is crucial for cellular defense against oxidative stress. To investigate whether the inhibition of Nrf2 affects the treatment of ASP, we utilized ML385, a specific Nrf2 inhibitor, to counteract the effects of Nrf2 [26]. The results indicated that the suppression of Nrf2 did not influence the expression of the PI3K/AKT pathway (Fig 5A–C). However, with the inactivation of Nrf2, its downstream related genes, such as GCLC, HO1, and GPX4, were downregulated compared to the ASP treatment group (Fig. 5D-F). Pretreatment with ML385 raised the levels of ROS in the AC16 cells (Fig. 5G and H). As anticipated, ML385 worsened cardiac injury in the ASP-treated group and offset the protective effect of PI3K upregulation (Fig 5I and J). In addition, the decreased red/green ratio of MMP indicated the inhibitory effect of Nrf2 inhibition on the mitochondrial protective properties of ASP (Fig 5K and L).

## Discussion

Nowadays, cancer is still one of the major diseases that threaten human health, and nearly 10 million people die from cancer every year [27]. As one of the most common drugs used for the chemotherapy treatment of cancer, DOX can induce a patient’s cardiotoxicity in a dramatically high percentage of 57%. Therefore, there is an urgent need to investigate the underlying mechanisms of DIC and to develop new and effective therapeutic drugs. The major mechanism of myocardial injury caused by DOX is that DOX can significantly raise ROS levels, which has been demonstrated in previous studies [28,29]. The excessive accumulation of ROS results in the impairment of cardiac myocyte’s mitochondrial function. In this study, we detected the -situation of mitochondria by using JC-1 staining which can reflect MMP, a critical indicator of mitochondrial integrity and function [30].

The results demonstrated that ASP has a great ability to reduce the production of ROS, which can effectively mitigate oxidative stress and subsequent damage to cellular components, including proteins, lipids, and chromosomes within cardiomyocytes. Excessive ROS also can result in the opening of mitochondrial permeability transition pores (mPTPs), leading to cell death, this is one of the main reasons for the imbalance of MMP in [31,32]. Our study utilized JC-1 staining to assess the injury level of mitochondria, the results showed that ASP could significantly rescue mitochondrial membrane potential, and this function may be accomplished by reducing intracellular lipid ROS.

When the cells are under conditions of oxidative stress, NRF2 is released following PI3K/AKT pathway activation, and then NRF2 will translocate from the cytoplasm to the nucleus and activate the downstream genes [33]. In the context of ferroptosis, NRF2 targets genes such as glutathione peroxidase 4 (GPX4), heme oxygenase 1 (HO1), and ferritin heavy chain (FTH1), which are crucial in mitigating oxidative stress and maintaining cellular iron homeostasis [21]. However, DOX can suppress the protein expression of the PI3K/AKT signaling pathway [24], which plays a vital role in cellular survival. The inhibition of this pathway by DOX also results in the downregulation of NRF2, a transcription factor that was important in the anti-oxidative system [34]. Consequently, this suppression impairs the myocardium’s ability to counteract oxidative stress, leading to the accumulation of lipid hydroperoxides and the induction of ferroptosis [35]. Our results demonstrate that ASP intervention leads to the activation of the PI3K/AKT/NRF2 pathway in DOX-treated cardiomyocytes. Activation of this signaling axis greatly raises myocardial survival, alleviates oxidative stress, and reduces myocardial injury markers such as ANP and BNP.

In summary, DOX-induced cardiotoxicity is associated with increased ROS production and mitochondrial dysfunction. The consequent dysregulation of PI3K/AKT/NRF2 exacerbates the cellular vulnerability to ferroptosis, highlighting the importance of the PI3K/AKT/NRF2 pathway in the pathogenesis of DOX-induced cardiotoxicity and the potential therapeutic targets for mitigating this effect.

The implications of our findings are profound, suggesting that ASP could serve as an adjunct therapy in cancer treatment protocols to minimize DOX’s cardiotoxic effects. This could potentially improve treatment outcomes and patient quality of life by reducing the incidence of chemotherapy-related heart disease.

While our in vitro findings are promising, they warrant further validation in preclinical models and clinical trials. Future studies should also explore the long-term effects of ASP on cardiac function and its potential synergistic effects when co-administered with other chemotherapeutic agents.

In conclusion, our study delineates mechanistic insights into how ASP could protect against mitochondrial dysfunction in cardiomyopathy. By targeting the PI3K/AKT/NRF2 pathway, ASP may offer a novel therapeutic strategy to combat the cardiotoxicity associated with DOX treatment.

## Supporting information

S1 Table The table provides qPCR primer sequences for human ANP, BNP, and GAPDH, used for gene expression analysis. (DOCX)

S1 Fig DOX-induced cardiotoxicity in AC16 and H9C2 cells. Flow cytometry was employed to detect DCFH-DA/FITC fluorescence in AC16 cells (A&C) and H9C2 cells (B&D), with statistical analysis performed on three replicates (n =  3); (E&F) The effects of DOX on the reduction of mitochondrial membrane potential (MMP) in AC16 cells were assessed using a red/green fluorescence ratio. A total of 150 cells were analyzed (scale bar =  100 μm); (G) Quantitative real-time PCR (q-PCR) was conducted to analyze the mRNA expression levels of ANP and BNP in AC16 and H9C2 cells (n =  3). One-way ANOVA (Tukey post-test), means ±  SD. P >  0.05, nonsignificant (ns), *  P <  0.05, ** P <  0.01, *** P <  0.001, **** P <  0.0001.
(TIFF)
