# Supplementary material for: Astragalus polyphenols attenuates doxorubicin-induced cardiotoxicity by activating the PI3K/AKT/NRF2 pathway
Source: PLoS One. 2025 Feb 25;20(2):e0319067. doi: 10.1371/journal.pone.0319067 (PMC11856579; doi:10.1371/journal.pone.0319067)
Supplement: S1 Table — (DOCX) [file pone.0319067.s001.docx]

**Supplementary Table S1. qRT-PCR primers used in the study.**

| Genes | Sense (5’-3’) | Antisense (5’-3’) |
| --- | --- | --- |
| GAPDH | GTCTCCTCTGACTTCAACAGCG | ACCACCCTGTTGCTGTAGCCAA |
| ANP | ACAATGCCGTGTCCAACGCAGA | CTCCTTCTGCATCCTGTCAGC |
| BNP | TCTGGCTGCTTTGGGAGGAAGA | CCTTGTGGAATCAGAAGCAGGTG |
