# Supplementary figures and images for: Astragalus polyphenols attenuates doxorubicin-induced cardiotoxicity by activating the PI3K/AKT/NRF2 pathway
Source: PLoS One. 2025 Feb 25;20(2):e0319067. doi: 10.1371/journal.pone.0319067 (PMC11856579; doi:10.1371/journal.pone.0319067)

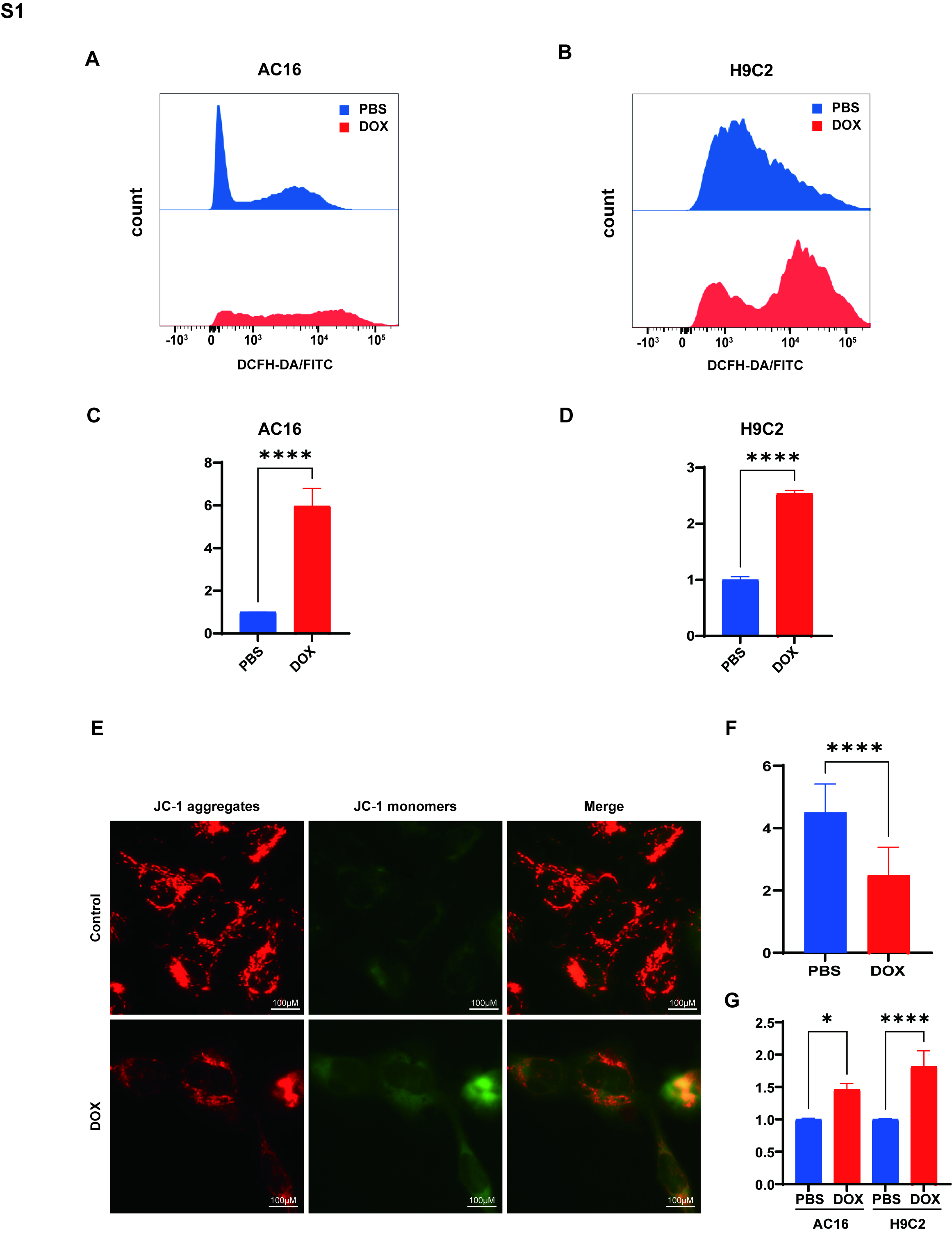

Supplement: S1 Fig — Flow cytometry was employed to detect DCFH-DA/FITC fluorescence in AC16 cells (A&C) and H9C2 cells (B&D), with statistical analysis performed on three replicates (n = 3); (E&F) The effects of DOX on the reduction of mitochondrial membrane potential (MMP) in AC16 cells were assessed using a red/green fluorescence ratio. A total of 150 cells were analyzed (scale bar = 100 μm); (G) Quantitative real-time PCR (q-PCR) was conducted to analyze the mRNA expression levels of ANP and BNP in AC16 and H9C2 cells (n = 3). One-way ANOVA (Tukey post-test), means ± SD. P > 0.05, nonsignificant (ns), * P < 0.05, ** P < 0.01, *** P < 0.001, **** P < 0.0001. (TIFF) [file pone.0319067.s002.tif]
